# Zero-Covid Strategy: What’s Next?

**DOI:** 10.34172/ijhpm.2022.6757

**Published:** 2022-01-25

**Authors:** Zhiqing Zhan, Jie Li, Zhangkai J. Cheng

**Affiliations:** ^1^Department of Allergy and Clinical Immunology, Guangzhou Institute of Respiratory Health, State Key Laboratory of Respiratory Disease, National Clinical Research Center of Respiratory Disease, First Affiliated Hospital of Guangzhou Medical University, Guangzhou, China.; ^2^Department of Clinical Medicine, The Third Clinical School, Guangzhou Medical University, Guangzhou, China.

## Introduction

 The goal of the strategy of control and maximum suppression (Zero-Covid) is to reduce virus transmission to near-zero levels and ultimately eliminate the virus within a specified geographic region.^1^ “Zero-Covid” means effective control of limited cases and zero transmission, that is, zero uncontrolled cases.^2^ Countries including South Korea, China, Vietnam, Australia, and New Zealand have all made or are making it a priority to contain and eliminate coronavirus disease 2019 (COVID-19). Singapore has recently stated that it will relax quarantine measures in two stages and advancing the “living with covid” policy.^3^ New Zealand also announced in early October that it will open its borders in four steps and sought to coexist with the virus and control its spread as the vaccination rate increased.^4^

 The COVID-19 global pandemic differs from the influenza pandemic that it has lasted 20 months.^5^ COVID-19 is also different from severe acute respiratory syndrome (SARS), which was completely eliminated after eight months of epidemic.^6^ The dominant Delta strain has now spread rapidly over the world, posing a threat to herd immunity plans.^7^ The immunity against SARS coronavirus 2 (SARS-CoV-2) that mankind has developed is now potent enough to exert evolutionary pressure, forcing the virus to adapt and mutate even more.^8^ As the viruses continue to replicate and divide, they might adapt to the human body with reduced severity, or they might evolve to be more lethal. Meanwhile, with the resumption of free movement of people and goods across borders in the next 2-3 years, countries that previously implemented a Zero-Covid strategy may face long-term import risk from regions of high prevalence. As a result, future implementation of a Zero-Covid strategy may need to account for this new trend in a prolonged pandemic.

## Why Should Zero-Covid Strategy Be Considered Adjusted?

###  Differences Between Epidemiological Characteristics of SARS-CoV and SARS-CoV-2

 The Zero-Covid strategy’s primary goal is to totally block SARS-CoV-2 transmission via using containment methods similar to those used to stop SARS-CoV.^1^ However, COVID-19 is quite different from SARS in terms of symptoms and incubation period. SARS-CoV infection results in more severe and characteristic clinical symptoms without transmissible incubation period. This enables swift identification and isolation of SARS patients and their close contacts. COVID-19 has a longer incubation period (median: 6.4 days) than SARS (median: 4.6 days) during which it does not cause clinical symptoms. The proportion of asymptomatic and moderate can be as high as 80%.^9^ Patients who are latent, mild, or asymptomatic can also transmit the virus, but they are difficult to diagnose, monitor, or isolate, and up to 59% of new cases are transmitted from these groups,^10^ which makes it difficult to entirely disrupt the transmission chain, rendering the containment of COVID-19 more challenging than SARS.

###  What Delta Variant Means for Zero-Covid Strategy?

 Delta variant has now become the most widespread mutant strain worldwide.^7^ R_0_ of the SARS-CoV-2 original strain was approximately 2.5, similar to that of SARS-CoV.^11^ However, R_0_ of the Delta variant has shot up to 5-9 and the transmission was dramatically boosted,^12^ significantly increasing the number of close contacts for COVID-19. Furthermore, Delta variant’s ability to escape immunity increases the risk of reinfection. Consequently, control measures that were effective against the original strain may no longer be effective against the Delta variant. If more close contacts are further isolated and tougher control mechanisms are implemented, the load on society and economy may be unbearable.

 In addition to social prevention and control, vaccination is the most efficient tool against COVID-19. Each type of vaccine is now less than 80% effective against the Delta variant globally, and its long-term efficiency remain uncertain. Additionally, breakthrough infections occur to some vaccinated individuals, making them carry equal viral loads and are equally likely to pass on their infections as in the unvaccinated.^7^ As vaccination rates improve, vaccine breakthrough cases are projected to increase. This means that, while widespread vaccination can significantly reduce the risk of hospitalization and severe disease, it cannot completely break the chain of transmission of COVID-19. It becomes exceedingly difficult to avert the pandemic by instilling population immunity through mass vaccination. The effectiveness of a selected group of widely used vaccines against SARS-CoV-2 and its variants are listed in Table.

**Table:** Summary of the Effectiveness of COVID-19 Vaccines

**Vaccine**	**Doses**	**Severity of Illness**	**Variant Not Specified**	** Ancestral Type (D614G)**	**Alpha (B.1.1.7)**	**Beta (B.1.351)**	**Gamma (P.1)**	**Delta (B.1.617.2)**
Pfizer-BioNTech	1	Infection	85% (69-93%)	UK SIREN (Day 21+): 72% (58-86%) Israel SHEBA (Day 15-28): 75% (72-84%) Israel Clalit (Day 14-20): 46% (40-51%)				
		Asymptomatic			Scotland: 38% (29%-45%)	American: 17% (10%-23%)		Scotland: 30% (17-41%)
		Symptomatic		Trial (Day 0-21): 52% (29.5%-68.4%) Israel SHEBA (Day 15-28): 85% (71%-92%) Israel CLALIT (Day 14-20): 57% (50%-63%) Israel Maccabi (Day 13-24): 51.4% (-7.2%-78.0%) England (Day 28+, 80+ years): 57% (48%-63%)	Scotland: 27% (13%-39%)	Cananda: 43% (22%–59%)^a^		Scotland: 33% (15%-47%)
		Hospitalization/ severe disease		Trial (severe disease): 100% (-52%-100%); Israel CLALIT (severe disease; Day 14-21): 62% (39%-80%)	England: 83% (62%-93%)	American: 0% (0%-19%)	Cananda: 56% (-9%-82%)	England: 94% (46%-99%); Canada (severe disease): 78% (64%-87%)
	2	Infection	94% (82-98%)	UK SIREN: 86% (76%-97%); Israel CLALIT: 92% (88%-95%)	Qatar: 89.5% (85.9%-92.3%)	Qatar: 75% (70.5%-78.9%)		
		Asymptomatic	94.4% (93.3-95.3%)		Israel National: 91.5% (90.7-92·2%)	American:75% (71-79%)		Scotland: 79% (75%-82%)
		Symptomatic	England: 93.3% (85.8%-96.8%); England: 89% (85%-93%); Israel: 98% (97.6%-98.3%); Trial (multinational, 4 months follow-up): 83.7% (74.7%-89.9%)	Trial (original): 94.6% (90.3%-97.6%); Trial (updated): 91.3% (89.0%-93.2%); Israel CLALIT: 94% (87%-98%); England (80+ years): 88% (84%-90%); Canada: 92% (96%-86%)	Israel national: 97.0% (96.7%-97·2%), Scotland: 92% (90%-93%), UK: 93.7% (91.6%-95.5%), Canada: 89% (87%-91%)	Trial (South Africa): 100% (53.5%-100.0%); Canada: 85% (70%-93%)^a^	Canada: 85% (70%-93%)^a^	Scotland: 83% (78%-87%); UK: 88% (85.3%-90.1%); Canada: 85% (59%-94%)
		Hospitalization/severe disease	Israel national: 97.5% (97.1%-97.8%); Israel (hospitalization): 98.2% (97.5%-98.7%); Israel (death): 98.5% (97.4%-99.2%)	Trial (severe disease): 75% (-152.6%-99.5%); Israel CLALIT (severe disease): 92% (75%-100%); Canada: 97% (79%-100%)	Qatar: 100.0% (81.7%-100.0%); UK: 95% (78%-99%); Canada: 96% (93%-98%)	Qatar: 100% (73.%-100%); Canada: 98% (82%-100%)^a^	Canada: 98% (82%-100%)a	UK: 96% (86%-99%)
Oxford–AstraZeneca	1	Asymptomatic			Scotland: 37% (32%-42%)		Brazil: 33% (32%-34%)	Scotland: 18% (9%-25%)
		Symptomatic		Canada: 78% (41%-92%)	Scotland: 39% (32%-45%)	Canada: 50% (27%-66%)^a^		Scotland: 33% (23%-41%); Canada: 70% (46%-83%)
		Hospitalization/severe disease			England: 76% (61%-85%)	Canada: 82% (61%-92%)^a^	Brazil: 52% (50%-53%)	England: 71% (51%-83%); Canada: 87% (56%-96%)
	2	Infection		Trial (UK): 51.9% (42.0%-60.1%)				
		Asymptomatic			Scotland: 73% (66%-78%)		Brazil: 70% (69%-71%)	Scotland: 60% (53%-66%)
		Symptomatic	Trial (UK, SA, Brazil): 66.7% (57.4%-74.0%); England: 78% (69.7%-84%); Trial (South Africa): 21.9% (-49.9%-59.8%)	Trial (UK): 74.2% (65%-81%)	Trial (variant-specific): 74.6% (41.6%-88.9%); Scotland: 73% (66%-78%); UK: 74.5% (68.4%-79.4%); Canada: 75% (-98%-97%)	Trial (South Africa): 10.4% (-76.8%-54.8%)	Brazil:78% (69%-84%); Trial (Brazil standard dose, non-variant-specific): 57.6% (40.7%-69.7%)	Scotland: 61% (51%-70%); UK: 67% (61.3%-71.8%)
		Hospitalization/severe disease			UK: 86% (53%-96%); Canada (≥7 days after dose 2): 67% (-155%-96%)		Brazil: 87% (85%-88%)	UK: 92% (75%-97%)
Novavax	2	Symptomatic	Trial phase 3 UK (7 days after dose 2): 89.7% (80.2%-94.6%)	Trial (UK): 89.3% (75.2%-95.4%)	Trial (variant-specific): 86% (59.2%-95.0%)	Trial (SA): 51.0% (-0.6%-76.2%)		
		Hospitalization/severe disease			UK: 100%			
Janssen	2	Asymptomatic	65.5% (39.9%-81.1%)					
		Symptomatic	Trial (USA, SA, Brazil): 66.1% (55.0%-74.8%)	Trial (USA): 72.0% (58.2%-81.7%)		Trial (SA): 64.0% (41.2%-78.7%)	Trial (Brazil): 68.1% (48.8%-80.7%)	
		Hospitalization/severe disease		Trial (USA): 85.9% (-9.4%-99.7%)		Trial (SA): 81.7% (46.2%-95.4%)	Trial (Brazil): 87.6% (7.8%-99.7%)	
Moderna	1	Infection	88% (61%-96%)	Trial (Day 0-21): 89.6% (85.2%-92.6%)				
		Symptomatic			Canada: 61% (56%-66%)	Canada: 78% (60%-88%)^a^		Canada: 70% (52%-81%)
		Hospitalization/severe disease			Canada: 59% (39%-73%)	Canada: 94% (75%-99%)^a^		Canada: 95% (67%-99%)
	2	Infection	84% (31%-96%)					
		Symptomatic		Trial: 94.1% (89.3%-96.8%); Canada: 91% (64%-98%)	Canada: 91% (84%-95%); Qatar: 100% (91.9%-100%)	Canada: 88% (61%-96%)^a^; Qatar: 96.4% (91.9%-98.7%)	Canada: 88% (61%-96%)^a^	
		Hospitalization/severe disease		Trial: 100% (NE); Canada (≥7 days after dose 2): 96% (85.3%-90.1%)	Canada: 94% (97%-90%)	Canada: 100%^a^		
CoronaVac	1	Asymptomatic					Brazil: 16% (15%-17%)	
		Symptomatic						China: 14% (-60%-55%)
		Hospitalization					Brazil: 27% (25%-28%)	
	2	Asymptomatic					Brazil: 54% (53%-55%)	
		Symptomatic	Trial (Turkey): 83.5% (65.4%-92%); Chile: 65.9% (65.2%-66.6%)	Trial (Indonesia): 65.3% (25 cases); Trial (Turkey): 91.3% (29 cases)			Trial (Brazil): 50.3% (252 cases: 167 placebo, 85 vaccine) Brazil (healthcare workers): 36.8% (-54.9-74.2%)	
		Hospitalization/severe disease	Trial (Turkey, severe disease): 100% (20.4%-100%); Chile (hospitalization): 87.5% (86.7%-88.2%); Chile (death): 86.3% (84.5%-87.8%)				Brazil: 73% (72-74%)	China: 59% (16-82%)

Abbreviation: COVID-19, coronavirus disease 2019. ^a^Results combined for both Beta and Gamma variant.

###  Socioeconomic and Livelihood Considerations

 While the Zero-Covid strategy can significantly reduce virus transmission and alleviate pressure on the healthcare system in the short term, long-term strict measures such as lockdown may disrupt society’s normal order, including the economy, personal employment, and public mental health.^2,3^ A survey showed that more than 50% of participants reported having some professional loss in the pandemic lockdown phase and 74% of the respondents reported having stress about their business or employment in the coming times.^13^ Zero-Covid strategy necessitates strict adherence by the people, and its effectiveness is contingent upon the public will.^1^ However, as the social and economic costs of implementing the control policy continue to rise, public support for and cooperation with the strategy may dwindle. When a country achieves a low mortality rate through widespread vaccination, it may be beneficial to consider adjusting the original pandemic prevention measures, establishing a dynamic balance between public health and normal social and economic order, and formulating a realistic long-term strategy.

## Factors to Be Considered

###  Vaccination Rate and Efficacy

 Asia is home to the majority of countries that have adopted a Zero-Covid strategy. Based on the COVID-19 R0 of 2.28 in Asia, for vaccines that have 70% and 80% efficacy rates, vaccination rates of 80.2% and 70.2%, respectively, are required to achieve herd immunity. To August 30, 2021, the vaccination rates of countries that are implementing or have implemented Zero-Covid strategy are Singapore 83%, China 80%, South Korea 55%, New Zealand 47%, Thailand 33% and Vietnam 17%.^14^ Given the increased transmissible power of the mutant strain, even if the vaccine is 100% effective, more than 83% of the population needs to be vaccinated for herd immunity to develop at an R0 of 6. However, the current maximum efficacy of vaccines against Delta is only about 76% (Moderna) with uncertain long-term efficacy, making herd immunity a long way off in some of these countries. Globally, vaccination rates in low-income countries are generally low, and more help should be given to form global herd immunity.^14^

###  Population, Medical Resources, and a Country’s Need for the Levels of Openness

 The population size of each country, the total quantity of medical resources available, and how those resources are distributed throughout the country must all be carefully studied. China has been following a Zero-Covid strategy and has been able to contain the outbreak in a pretty short period of time, with death toll hitting only 5681 as of this writing.^15^ This is the result of China’s concentration of national medical resources and large treatment costs.^16^ Given China’s vast population (over 1.4 billion), density, and uneven distribution of medical resources among regions, if mitigation strategy was taken, the country would struggle to keep the number of patients within the system’s capacity as infection rates grow.

 What strategy to choose is also determined by a country’s need for the levels of openness. A lengthy blockade of an open economy that is heavily reliant on international trade, business, and tourism would be devastating to its economy. But if the same approach is followed in other nations with sizable domestic markets that can support their own commerce and tourism, impact will be much eased. As a result, policymakers must strike a delicate balance between the economy’s long-term viability, family livelihoods, and public health.

## What Comes Next?

###  Accelerate Vaccination and Vaccine Research and Development

 With the push for mass vaccination, most of diagnosed cases can now be isolated at home and recovering on their own, thanks to a marked drop in severe illness and mortality, thus alleviating the demand for medical resources. This allows governments to focus on severely ill patients while easing up on certain pandemic prevention measures. Devise realistic steps are needed to boost vaccination rates. We advise governments to identify causes of opposition to universal vaccination, such as vaccination safety concerns, and to communicate openly to foster public trust. Vaccination may also be included in the requirements for long-distance travel, employment, and education, with exceptions granted for selected populations who cannot be vaccinated due to medical reasons. Vaccine hesitation and disinformation are also important reasons for the low willingness to vaccinate. Government departments should work with social media to vigorously control the spread of false and misinformation. At the same time, the government needs to speed up the research and development of new vaccines, develop booster shots for each mutant strain, and study the actual effectiveness of a combination or sequential vaccination of multiple vaccines. In addition, future vaccine development should also consider the elderly, patients with underlying diseases, immune deficiency diseases, and other key protection groups.

###  Paying Attention to Tackling the Inequities and Protecting the Vulnerable Groups

 It is critical to accompany any restrictions with adequate resources to address unmet needs.^17^ For example, people who cannot work remotely may have a higher risk of infection with COVID-19, so it is important to provide them with necessary support resources, such as safe isolation space, N95 masks, paid sick leave, etc. Public health should design services allocating greater investments to mitigate the structural inequities and to address the unmet needs.

 Although the disease burden affects people in every class, it seems to have a greater negative impact on the poor and vulnerable groups.^18^ For example, people with low socio-economic status tend to live in crowded places and take public transport, which increases the risk of infection and virus transmission to their family members and communities. In addition, due to unemployment, low-income and low educational level, etc, the group is less likely to seek treatment and health services, which eventually may lead to higher incidence rate and mortality.^18^ Governments must take steps to combat this disease in vulnerable groups specifically, such as legislating long-term laws to improve social welfare and pay attention to insurance along with livelihood assistance, reimbursing and compensating for the lost income of the affected strata, providing essential health services and allocating support packages to high-risk and vulnerable people.

###  Establish a Reasonable Safety Margin and Dynamically Modify it in Stages

 The opening of economic and social activities and borders should be carried out gradually, and dynamically modified in response to changes in the pandemic situation at home and abroad, under the premise that the pandemic is controlled and the medical system is capable of bearing the burden of the pandemic. We suggest governments design emergency medical resource allocation plans and cross-regional resource allocation plans after accurate evaluation of medical resources in this region. At the same time, the economic and social costs that may be brought about by different levels of pandemic control measures should be refined and the scale of pandemic control should be determined to prevent the exhaustion of medical resources and disruption of social order, and a reasonable margin of safety should be set accordingly. That is, what level of control measures should be activated/removed under what conditions, and how to set the intensity, duration and scope of control, etc, so as to gradually make the public adapt to it through dynamic adjustment in stages. At the same time, the government should integrate COVID-19 testing into daily routines and establish an effective contact tracing system. It is also recommended that governments conduct a national serum antibody test before adjusting their strategy.

###  Reach a New Consensus on Pandemic Prevention Expectation With the Public

 Adjusting the pandemic prevention target requires building a new societal consensus, lowering expectations of state intervention in COVID-19 measures to foster public cooperation. It is suggested that governments, while releasing the latest pandemic prevention policies, interpret pandemic data through press conferences and media, and defuse public anxiety and fear.

## Conclusion

 At present, the global COVID-19 pandemic is still developing. We recommend countries to formulate a long-term strategy that is realistic for countries based on their specific circumstances. Policy-makers should pay attention to tackling the inequities and protecting the vulnerable groups. Rational adjustment of the Zero-Covid strategy based on the premise of manageable disease burden help transition to a more manageable state, strike a dynamic balance between public health and normal social and economic order.

## Ethical issues

 Not applicable.

## Competing interests

 Authors declare that they have no competing interests.

## Authors’ contributions

 Conception and design: ZZ; Administrative support: ZJC; Manuscript writing: All authors; Final approval of manuscript: All authors.

## Funding

 This study was supported by the Guangzhou Institute of Respiratory Health Open Project (grant number 2020GIRHHMS04), and the Zhongnanshan Medical Foundation of Guangdong Province (grant number ZNSA-2021005).
